# Is Narrative Comprehension Embodied? An Exploratory Study on the Relationship Between Narrative and Motor Skills in Preschoolers

**DOI:** 10.3390/children12080999

**Published:** 2025-07-29

**Authors:** Emanuele Di Maria, Raffaele Dicataldo, Maja Roch, Valentina Tomaselli, Irene Leo

**Affiliations:** Department of Developmental Psychology and Socialization, University of Padua, 35131 Padua, Italy; emanueledimaria97@gmail.com (E.D.M.); raffaele.dicataldo@unipd.it (R.D.); valentina.tomaselli@phd.unipd.it (V.T.); irene.leo@unipd.it (I.L.)

**Keywords:** narrative comprehension, motor skill, cognitive skills, language abilities, embodied cognition

## Abstract

**Background/Objectives:** According to Embodied Cognition theories, motor skills in early childhood are closely interconnected with various cognitive abilities, including working memory, cognitive flexibility, and theory of mind. These processes are integral components of the multicomponent model of narrative comprehension, which posits that higher-order cognitive functions support the construction of coherent mental representations of narrative meaning. This study aimed to examine whether motor skills directly contribute to narrative comprehension in preschool children or whether this relationship is mediated by cognitive skills. **Methods:** Seventy-four typically developing children aged 3 to 6 years (47.2% female) participated in this study. Motor skills were assessed using standardized measures, and cognitive abilities were evaluated through tasks targeting working memory, cognitive flexibility, and theory of mind. Narrative comprehension was measured with age-appropriate tasks requiring the understanding and retelling of stories. A structural equation model (SEM) was conducted to test the direct and indirect effects of motor skills on narrative comprehension via cognitive skills. **Results:** The SEM results indicated a significant direct effect of motor skills on cognitive skills and an indirect effect on narrative comprehension mediated by cognitive abilities. No evidence was found for a direct pathway from motor skills to narrative comprehension independent of cognitive processes. **Conclusions:** These findings underscore the complex interplay between motor, cognitive, and language development in early childhood. The results suggest that motor skills contribute to narrative comprehension indirectly by enhancing core cognitive abilities, offering novel insights into the developmental mechanisms that support language acquisition and understanding.

## 1. Introduction

Narratives play a fundamental role in children’s earliest language experiences, contributing significantly to the development of communication and language skills, as well as preparing them for school. Narrative comprehension refers to the process through which lexical information, sentences, and other types of input are interpreted, enabling the creation of a coherent mental representation of a narrative’s meaning [1]. As [2] argued, narratives offer a privileged context in which children learn to connect events over time, establish coherence, and attribute mental states to characters. Compared to routines or expository texts, narratives engage a broader range of cognitive processes, including perspective taking, inference generation, and the management of causal chains, making them particularly suitable for investigating the interaction between language, cognition, and motor skills during the preschool years.

This study is grounded in the theoretical framework of the multicomponent model for comprehension [1,3,4]. According to this model, narrative comprehension arises from the dynamic and reciprocal interaction between language and cognitive skills. This process encompasses a range of abilities, broadly categorized into two groups: lower-order language skills and higher-order cognitive skills, which operate at two distinct levels of processing [1]. The first processing level comprises components essential for basic linguistic information processing, including vocabulary and grammar. The second level encompasses components necessary for achieving a coherent global representation of the text’s meaning, such as inferential abilities, integration of prior knowledge, understanding of story structure, executive control, and theory of mind. While the multicomponent model has been tested in both preschool and school-aged children, there are still some components that were not included, notably motor skills. There is growing evidence suggesting that during development, motor skills support language development in different ways. This is conceptualized within the Embodied Cognition framework, which posits that all cognitive processes, the including linguistic ones, are deeply rooted in the body’s interactions with the environment [5,6].

This exploratory study aims to investigate direct and indirect pathways involving motor skills, language, and cognitive abilities within the framework of the multicomponent model of narrative comprehension.

### 1.1. Relationship Between Motor Skills and Language Development and Narrative Competence

Motor skills involve controlling muscle movements and include gross motor skills, which use large muscle groups for activities such as walking, running, jumping, and maintaining balance; and fine motor skills, which use smaller muscles, particularly in the hands and fingers, for tasks including writing, buttoning clothes, or using utensils [7]. Both gross and fine motor skills develop from early childhood and are crucial for everyday but also for cognitive tasks. The theoretical perspective of Embodied Cognition suggests that our understanding of concepts and language is shaped by our experiences [5,6]. Over the years, motor skills have received significant attention for their critical role in language acquisition. Consistent correlations have been found between language acquisition and various sensorimotor components, with both gross motor skills (GMSs) and fine motor skills (FMSs) showing a significant relationship with language development. Research examining this relationship in infants has highlighted significant connections between the two domains, particularly within the first two years of life [8,9,10]. While fewer studies focus on this link in preschool-aged children, recent research has found relationships between motor skills and various aspects of language, including vocabulary [11,12] and grammar [13,14].

More recently, this motor–language relationship has been examined in the context of narrative competence—a higher-order language skill. Narrative comprehension involves constructing a coherent mental model of story content through the transformation of textual information into meaningful representations [15]. According to the Embodied Cognition framework, this meaning-making process may also be grounded in the child’s bodily and motoric experiences. Supporting this view, recent research has found that motor skills are positively associated with preschoolers’ ability to comprehend and produce coherent narratives [16,17], suggesting that the body may play an important role in how young children understand stories.

### 1.2. The Role of Cognitive Skills in the Relationship Between Motor Skills and Narrative Competence

The connection between motor skills and narrative competence is grounded in brain development, where motor experiences—such as gestures and object manipulation—engage sensorimotor systems that also support cognitive functions such as memory and attention [18]. These embodied processes help children organize and sequence events, thereby fostering narrative competence. Within this developmental framework, cognitive skills may act as mediators, particularly those involved in both motor and narrative domains from early childhood. This study focuses on working memory, cognitive flexibility, and theory of mind (ToM), given their established roles in narrative comprehension. Working memory enables the retention and integration of information across sentences and events and is essential for maintaining coherence and making inferences within stories [19,20]. Empirical studies have consistently shown that higher verbal and visuospatial working memory predicts better narrative understanding in preschoolers [1,21,22]. Moreover, motor abilities have been linked to the development of working memory, suggesting a close connection between physical and cognitive growth [23]. Cognitive flexibility, the ability to shift between mental sets and perspectives, supports narrative comprehension by allowing children to adapt to changing story elements and integrate diverse viewpoints [24]. This ability also supports shifting between local and global narrative structures [25]. It is positively associated with motor development, particularly in tasks requiring balance, coordination, and fine motor control [26,27], with stronger effects observed in preschoolers [23]. Alongside these skills, ToM—the ability to attribute mental states to oneself and others [28]—is crucial for understanding characters’ beliefs, goals, and intentions, thereby supporting causal reasoning and coherence in narrative structure [29,30]. Recent studies indicate that motor experiences may also contribute to ToM development, particularly through interactive activities such as group play and structured storytelling involving explicit discussion of mental states [31]. These findings support the rationale for examining both direct and indirect pathways from motor skills to narrative comprehension, with cognitive abilities acting as potential mediators—an approach adopted in the present study.

## 2. Aim

The primary aim of this study was to examine the connections between these skills. In addition to evaluating the role of language skills (specifically morphosyntactic abilities) in narrative comprehension between the ages of 3 and 6, we addressed this relationship through two main research objectives:

1. To examine the direct effects of motor skills on narrative comprehension, as well as on cognitive abilities, including working memory, cognitive flexibility, and theory of mind.
2. To explore whether the relationship between motor skills and narrative comprehension is partly accounted for by indirect associations through cognitive abilities.

Building on previous research grounded in the Embodied Cognition framework [16,17] and the multicomponent model of narrative comprehension [1,3,4], we hypothesize that motor skills will exhibit a direct and significant relationship with both cognitive and narrative skills. This study’s innovation lies in examining the potential indirect relationship between motor and narrative skills, mediated by cognitive skills. We expect this indirect relationship to be statistically significant, highlighting the role of cognitive skills in linking motor abilities to narrative comprehension.

## 3. Materials and Methods

### 3.1. Participants

We recruited 74 children (47.2% girls) aged 3–6 years (mean: 54.69 months; 10.21 SD), all of whom were typically developed (TD) monolinguals, from a single preschool in a neighborhood of a medium-sized town in northern Italy XXX. The project was voluntary, and all parents enrolled voluntarily agreed to allow children to take part in the project. Parents provided written informed consent, agreeing to participate in the research. The tasks were administered in a predetermined sequence, following the recommended approach for studying individual differences (as suggested by [32]). This study was approved by the Ethics Committee of the School of Psychology at the University of Padova (protocol number 3141) and performed in accordance with the principles expressed in the 1964 Declaration of Helsinki.

### 3.2. Materials

#### 3.2.1. Narrative Comprehension

Narrative comprehension was assessed through the MAIN (Multilingual Assessment Instrument for Narratives) task, specifically the Italian version by [33]. This test consists of two sections. The first section involves presenting children with a series of sequential pictures where the children will have to tell the story. The examiner provided standard instructions such as “Look at these pictures carefully. They tell a story. Tell me everything that happens.” During this phase, no feedback was given about the content of the narration, but minimal encouragement (e.g., “Can you tell me more?”) was allowed if the child stopped talking prematurely. The child’s narrative was audio-recorded and later transcribed verbatim. After seeing the pictures and telling the story, children answer 10 questions designed to assess their understanding of the narrative, including identifying key events, understanding causes and effects, and making inferences, with a maximum score of 10. Each correct answer received 1 point, following the scoring guidelines provided in the MAIN manual.

This section is specifically designed to evaluate text comprehension and global narrative structure. Children are asked open-ended questions that assess their understanding of the characters’ goals, causal links between events, and story resolution. The responses are scored by trained assessors using standardized coding criteria and prototypical answers provided by the MAIN protocol. Each response is evaluated for its coherence and relevance to the narrative, yielding a quantifiable measure of macrostructural comprehension. This approach allows us to examine children’s ability to construct a coherent mental representation of the story—what the editor refers to as understanding “large segments.” Each correct answer received 1 point, following the scoring guidelines provided in the MAIN manual. The test includes four culturally neutral stories, each containing six pictures, ensuring consistency of cognitive and linguistic complexity. Although not yet standardized, the MAIN follows standardized procedures, making it suitable for scientific and assessment use. For our study, we used the story “The Little Goats”. This story involves a sequence in which a group of young goats and their mother are near a body of water, and while the mother goat is occupied rescuing one of the kids, a hidden fox emerges and seizes another goat. A bird observing the scene intervenes to help, allowing for the assessment of causal reasoning, inferential understanding, and attribution of intentions and emotions. Examples of MAIN task questions include: ‘Why did the mother goat enter the water?’ and ‘Why is the fox jumping forward?’. The MAIN task has demonstrated high interrater reliability, with Cohen’s kappa values of 0.90 for the number of macrostructure elements in storytelling and 0.90 in retelling, as well as a kappa of 0.83 for mental state terms in storytelling, indicating its effectiveness as a reliable assessment tool in evaluating narrative competence in children [34].

#### 3.2.2. Motor Skills

Motor skills were assessed using the Peabody Developmental Motor Scales 2 (PDMS-2), Italian version by [7]. This comprehensive age-normed assessment includes tasks designed to evaluate both gross and fine motor skills. Gross motor skills were assessed through activities such as running, jumping, and balancing, while fine motor skills were evaluated through tasks involving grasping, object manipulation, and visuomotor integration. The maximum cumulative score obtainable is 514, reflecting a child’s overall motor competence. The tasks were scored in real time by trained assessors during administration, allowing for immediate and accurate evaluation of each child’s performance. Specifically, the Stationary subtest required children to maintain balance in various positions (e.g., standing on one foot for ≥5 s, standing on tiptoes without support, imitating body postures demonstrated by the examiner). The Locomotion subtest assessed dynamic gross motor skills through tasks such as walking in a straight line, running, galloping, hopping forward on one foot, jumping over a low obstacle, skipping (alternating feet), and performing a running kick. The Object Manipulation subtest included catching a football and a tennis ball, throwing underhand and overhand at a target, and kicking a football. Fine motor assessment included the Grasping subtest (e.g., picking up 1 cm beads, stacking eight 2.5 cm cubes) and the Visual–Motor Integration subtest, which required copying shapes (circles, crosses, squares) and tracing straight lines. All items were administered and scored according to the basal-and-ceiling rules specified in the PDMS-2 manual. At the conclusion of the assessment phase, scores were used to calculate two standard quotients: the Gross Motor Quotient (QGM) and the Fine Motor Quotient (QFM), each with a mean of 100 and a standard deviation of 15. PDMS-2 demonstrates excellent reliability (99%) and is validated as a robust tool for assessing motor development in children. These quotients allow for the comparison of different motor abilities and the identification of potential discrepancies between them. The Total Motor Quotient (QMT) is obtained by combining the QGM and QFM, providing a comprehensive index of overall motor skills. This test was scored in real time by the assessor.

#### 3.2.3. Language Skills

The “Sentence Completion” task from the BVL 4-12 (Language Assessment Battery) developed by [35] was used for assessing language skills. This task was included to assess morphosyntactic competence, which plays a key role in sentence-level processing and supports narrative comprehension by enabling children to understand and produce grammatically coherent utterances. This battery of standardized tests assesses language skills between 4 and 12 years. Specifically, sentence competition involves children listening to a complete sentence and then completing the beginning of a second sentence by assigning the appropriate morphemes to the verb, with increasing difficulty. The task specifically evaluates grammatical and syntactic skills, with a focus on understanding and using verb morphology. The maximum score for this task is 14, reflecting the child’s competence in sentence construction. For example, a child might hear: “*La mamma cucina. Le mamme…*” (The mother is cooking. The mothers…) and must complete the sentence with “*… le mamme cucinano*” (… mothers are cooking). The task has demonstrated high reliability, with internal consistency coefficients (Cronbach’s alpha) above 0.80, ensuring the precision of the language assessment [35].

#### 3.2.4. Cognitive Skills

Three separate age-normed tasks were used to evaluate distinct cognitive domains: the DCCS for cognitive flexibility, a visuo-spatial task for working memory, and the NEPSY-II theory of mind subtest as a proxy measure of early social-cognitive reasoning.

Cognitive flexibility was assessed using the Dimensional Change Card Sort (DCCS) task, developed by [36]. In this task, children were asked to sort cards featuring either a red rabbit or a blue boat into corresponding boxes based on different rules. Initially, children sorted the cards by color, placing the blue boats in the blue rabbit box and the red rabbits in the red boat box. In the next phase, they sorted the cards by shape, with boats being placed in the boat box and rabbits in the rabbit box. Finally, in the Borders Game phase, children had to combine the two rules. Cards with black borders were sorted by color, while those without borders were sorted by shape. This task measures the child’s ability to adapt to changing instructions and switch between different sorting criteria. The maximum score for the test is 24 points. The DCCS has demonstrated good reliability, with test–retest and internal consistency coefficients ranging between 0.80 and 0.90, depending on the children’s age and the testing context [36].

Theory of mind: To investigate early social-cognitive reasoning, we administered the Theory of Mind subtest from NEPSY-II (Italian edition by [37]). This standardized task is designed to tap into key components of theory of mind, such as the understanding of beliefs, desires, and intentions. In this task, children were presented with scenarios that required them to interpret and infer the mental states of others, including beliefs, desires, and intentions, which are fundamental for social cognition. The subtest includes both verbal and non-verbal items. Verbal tasks involve the examiner reading short stories or describing pictures, after which the child answers questions about what a character knows, thinks, or feels. Non-verbal items require the child to look at sequences of pictures and select the one that best completes the scenario based on the character’s mental state. The maximum achievable score on the test is 17 points. Examples of questions include “What does this child think is inside the box?” (assessing false belief) or “Which of these things does the child want?” Correct answers demonstrate the child’s ability to attribute beliefs and intentions to others. The NEPSY-II subtests, including Theory of Mind, show high reliability, with internal consistency coefficients generally exceeding 0.80 [37].

Working memory: Phonological working memory was assessed through the “Pappagallo Lallo” nonword repetition task developed by [38]. In this task, children were presented with a series of nonwords and asked to repeat them as accurately as possible. This task measures the child’s ability to temporarily store and manipulate verbal information. The maximum score obtainable on the test is 16 points. The Pappagallo Lallo task has demonstrated good reliability, with a Cronbach’s alpha of 0.78 [38].

### 3.3. Procedure 

Trained psychology graduate students assessed each child individually in a quiet room within the school. The battery of tests chosen was divided into three different sessions, each lasting 30 min. The distance among the sessions was not longer than 10 days to avoid, as much as possible, influencing this study’s outcomes due to child development factors. The order of tasks within each session was fixed to ensure consistency across participants. Given the age of the children, we aimed to create a comfortable and engaging research environment while maintaining their motivation. Specific trial-by-trial data-taking protocols were provided for each session.

## 4. Data Analysis

For this exploratory study, data analysis proceeded as follows. First, descriptive statistics were conducted to identify outliers and assess the overall abilities of the sample. This step is crucial to ensure the accuracy of subsequent analyses and to gain an initial understanding of the sample’s characteristics. Given the exploratory nature of this study, any missing data was removed to maintain data integrity and to avoid potential biases.

Next, correlation analyses were performed to examine the relationships between the variables. The aim was to detect any potential overlap between cognitive and motor skills, as well as other relevant constructs, which may suggest multicollinearity or shared variance. Additionally, given the broad age range in the sample, partial correlations controlling for age were conducted to ensure that the observed associations are not confounded by age differences. To control for age in the correlation analysis, a custom function to calculate the residuals for each variable, accounting for age as a predictor, was used. Specifically, for each variable, a linear regression was conducted with age as an independent variable. The residuals from these models, which represent the part of each variable not explained by age, were extracted. After obtaining these residuals for all key variables, a correlation matrix between them was computed. This approach ensures that the correlations reflect the relationships between the variables while controlling for the influence of age.

To address the primary research questions, structural equation modeling (SEM) and model comparisons were employed. SEM allows for the examination of both direct and indirect relationships between motor skills, cognitive skills, and narrative comprehension. The latent variable representing cognitive skills (CSs) was constructed by combining theory of mind (ToM), cognitive flexibility (CF), and phonological working memory (WM), which were assessed through standardized tasks. This latent variable captures the shared variance among these components, representing a comprehensive measure of cognitive functioning. Motor skills (MSs) and language skills (LSs), along with age (in months), are included as direct predictors of both CSs and narrative comprehension (NC). Age was entered as a covariate in all models to control for age-related differences in cognitive and motor development.

Although SEM is generally recommended for larger samples, especially to avoid estimation bias and convergence issues [39], recent guidelines emphasize that a small-sample SEM can be informative when models are well specified and based on strong theoretical foundations [40]. Accordingly, we retained our SEM approach while transparently acknowledging its limitations in light of sample size.

Data analysis was performed using the R tool 4.2.2 (2022-10-31 ucrt) in the R-Studio IDE. Before proceeding with the structural model analysis, it is useful to perform descriptive analyses and correlations between the variables under consideration.

## 5. Results

To compare children’s performance to the normative sample, it was necessary to convert raw scores (Table 1) into standard scores. As for motor skills, children obtained a mean standard value of 102.23. The mean value of the reference population is 104.16 ± 9.50 [7]. Therefore, the children performed within the average range of the normative sample for their age. As for cognitive flexibility, different means by age were calculated to allow comparison with the normative data. The performance of children aged 3–4 years was at the 17th percentile, indicating lower performance compared to the normative sample. The 4–5 years old children showed slightly above-average performance, standing at the 59th percentile, while those aged 5–6 years performed around the sample average, scoring at the 49th percentile. Regarding the working memory, children scored an average of 12.63 (range 0–16) correct repetitions, and this is to be considered an age-appropriate performance. Regarding ToM tasks, the children obtain a mean score of 5.93 (range 0–17), and this is considered an appropriate age performance [37]. Regarding narrative comprehension, the children obtained a mean score of 7 (range 0–10), and this is considered an appropriate performance compared to similar-age performance reported in previous studies [34]. Regarding sentence completion, the children obtained mean z-scores of 0.54 and consequently showed slightly above-average performance compared to the reference sample.

Narrative comprehension shows moderate correlations with cognitive flexibility, theory of mind, and working memory, with a range of correlations from 0.35 to 0.58. Motor skills are weakly correlated with theory of mind (0.26). The main predictors among them show moderate correlations, with theory of mind correlating strongly with various other variables, such as language skills (0.57), working memory (0.46), and cognitive flexibility (0.61). Overall, the correlations among the measured variables are of moderate degree, suggesting that there is a significant interdependence between narrative, motor, and cognitive skills.

A comparison between the correlations (Table 2) and partial correlations controlling for age (Table 3) shows changes in the relationships among the variables. Notably, the correlation between working memory and narrative comprehension becomes non-significant when age is controlled for, as does the correlation between cognitive flexibility and working memory. Overall, most relationships decrease in strength, except for the relationship between ToM and motor skills, which becomes stronger.

To validate the latent variable representing cognitive skills (CSs), we first performed a separate confirmatory factor analysis (CFA). The model fit indices demonstrated excellent fit (CFI = 1.000, TLI = 1.000, RMSEA = 0.000, SRMR = 0.000). The CS latent factor was specified based on three observed variables: working memory (WM), cognitive flexibility (CF), and theory of mind (ToM). All factor loadings were significant, with standardized estimates of 0.512 for WM, 0.692 for CF, and 0.891 for ToM, indicating strong associations with the latent construct.

Subsequently, we constructed a structural equation model (SEM) using the validated CS factor to examine how narrative comprehension (NC) is predicted by motor skills (MSs), age, language skills (LSs), and cognitive skills (CSs). The model fit indices ranged from acceptable to suboptimal (RMSEA = 0.179, CFI = 0.976, TLI = 0.835, SRMR = 0.030), and the model explained 52.9% of the variance in narrative comprehension.

**Figure 1:**
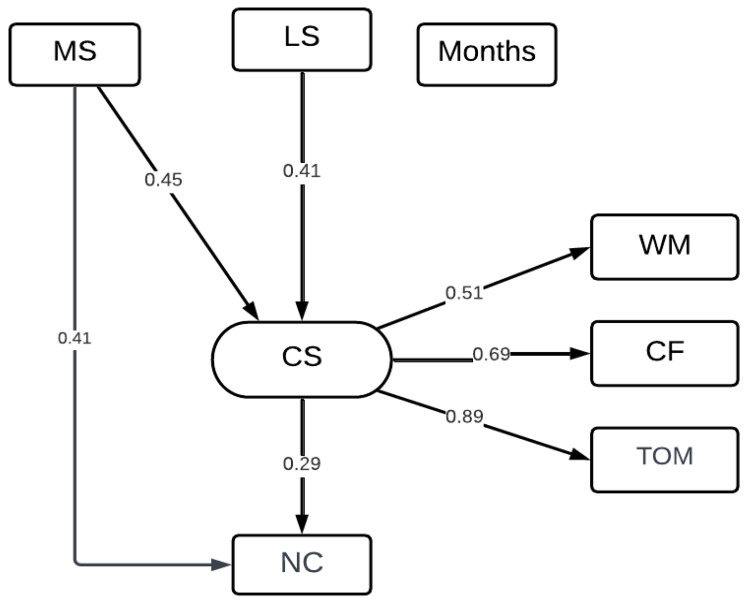
The significant pathway within the SEM. LSs = language skills; MSs = motor skills; WM = working memory; CF = cognitive flexibility; ToM = theory of mind; NC = narrative comprehension.

The results showed that motor skills significantly predicted narrative comprehension both directly (ß = 0.395, *p* = 0.009) and indirectly through cognitive skills (ß = 0.137, *p* = 0.041). Cognitive skills, in turn, significantly predicted narrative comprehension (ß = 0.294, *p* = 0.008), whereas age did not (ß = 0.132, *p* = 0.340). Regarding the predictors of cognitive skills, both motor skills (ß = 0.465, *p* = 0.001) and language skills (ß = 0.355, *p* = 0.001) had significant effects, while age was non-significant (ß = −0.017, *p* = 0.911) (Figure 1).

To further assess the robustness of our findings, we estimated an alternative SEM in which the latent construct of cognitive skills was defined directly within the structural model, integrating both measurement and structural components in a single step. This fully integrated model yielded improved fit indices (χ^2^ = 5.150, df = 9, *p* = 0.821; RMSEA = 0.000; CFI = 1.000; TLI = 1.056; SRMR = 0.026), suggesting a better approximation of the observed covariance structure. This model explained 50.4% of the variance in narrative comprehension.

To cross-validate these results using a more parsimonious specification and robust inference approach, we also estimated a structural model with bootstrapped standard errors (2000 resamples). This model, which included the executive function construct (based on working memory, cognitive flexibility, and theory of mind) defined directly within the SEM, showed results largely consistent with the previous analyses. Model fit was acceptable: χ^2^(2) = 4.75, *p* = 0.093; CFI = 0.967; SRMR = 0.042. However, the RMSEA (0.142) and TLI (0.885) indicated suboptimal fit, consistent with the model complexity and the small sample size. The pattern of associations was generally consistent with previous analyses: motor skills were significantly related to narrative comprehension, both directly and—albeit less robustly—indirectly through cognitive skills. Specifically, while the indirect effect remained in the expected direction, it no longer reached conventional significance levels (*p* = 0.073). These results suggest that the indirect effect of motor skills through cognitive skills may be more fragile and warrant further investigation in larger samples. Parameter estimates from the bootstrap model (Table 4) confirmed a significant effect of motor skills on both cognitive skills (est = 0.006, *p* = 0.002, 95% CI [0.002, 0.010]) and narrative comprehension (est = 0.010, *p* = 0.002, CI [0.004, 0.017]). Cognitive skills were also significantly associated with narrative comprehension (est = 0.558, *p* = 0.015, CI [0.146, 1.016]). The product term representing the indirect path was small (est = 0.003, *p* = 0.073), but its confidence interval [0.001, 0.008] did not include zero, indicating a consistent, though less robust, pattern of association.

## 6. Discussion

This study was designed as an exploratory investigation to assess the feasibility of modeling the relationships between motor skills, cognitive abilities, and narrative comprehension in preschool children. The primary aim was to generate preliminary evidence and identify potential pathways that could inform future, more rigorous research. Given the pilot nature of this study and the relatively small sample size, the findings should be interpreted as preliminary and not intended to support generalizable conclusions. Rather, they provide an initial framework and highlight key methodological considerations for subsequent studies, such as the need for replication with larger samples and more robust model validation procedures. 

Notably, no a priori power analysis was conducted, as the primary objective was to explore feasibility and identify methodological issues. Furthermore, due to the limited degree of freedom and sample size, parameter estimates should be interpreted with caution. Although an alternative model specification yielded improved fit indices, we retained the original SEM to maintain consistency with the theoretical framework. The alternative model is reported as supplementary evidence to support transparency and encourage further exploration.

The main objective of this study was to investigate the relationship between motor skills, language abilities, cognitive functions, and narrative comprehension in preschool children. In particular, we aimed to determine whether motor skills contribute to narrative comprehension within a multicomponent model, alongside age, language, and cognitive abilities. The primary finding of this study is that motor skills affect narrative comprehension both directly and indirectly through essential cognitive abilities, which, in turn, have a direct influence on narrative comprehension. These results align with previous research that has explored narrative comprehension within the framework of the multicomponent model [1,4]. Moreover, the findings demonstrate the contribution of an integrated set of linguistic and cognitive abilities in explaining individual differences in narrative comprehension [1,4], while also aligning with recent studies that have identified a relationship between motor skills and narrative comprehension [12,17]. What our results suggest in a preliminary way is the critical importance of integrating motor skills into multicomponent models, as they are pivotal components that contribute significantly to the cognitive processes underlying narrative comprehension in preschool children. By incorporating motor skills alongside linguistic abilities and cognitive skills, researchers can achieve a more comprehensive understanding of the intricate mechanisms involved in narrative comprehension. Our model explained the 52% variance in narrative comprehension, which, while consistent with the framework of the multicomponent model, is lower than the variance reported in other studies, such as [1], who reported an *R*^2^ of 0.60 and [4], who reported an *R^2^* of 0.85. One possible explanation for this difference is the preliminary nature of our study, which included a smaller sample size and a more focused set of predictors compared to previous models. Both [1,4] incorporated additional variables that likely contributed to the higher explanatory power of their models. For instance, they considered broader linguistic measures, including syntactic complexity and lexical diversity, and integrated literacy-related skills, such as phonological awareness and print knowledge, as well as a more comprehensive set of executive function components. Moreover, prior studies have investigated lower-order language skills, including vocabulary, as well as higher-order cognitive skills, such as inferential abilities, the integration of prior knowledge, and understanding of story structure. Executive control has also been examined in greater detail in previous research. Our study concentrated on linguistic skills, particularly morphosyntactic abilities, as well as cognitive skills, including theory of mind, working memory, and cognitive flexibility. Additionally, motor skills were introduced as a novel predictor within the multicomponent model of narrative comprehension. Future studies could enhance their scope by including additional linguistic and cognitive components, such as vocabulary, inferential abilities, and executive control, to investigate how these factors interact with motor skills in influencing narrative comprehension. Broadening the model to encompass a wider range of predictors while retaining a focus on motor skills may yield a more thorough understanding of the cognitive and linguistic processes involved.

Further research is needed to deepen our comprehension of the mechanisms underlying this relationship. First, it is necessary to divide fine and gross motor skills, as they might have different predictive power in relation to both cognitive skills and narrative comprehension. In fact, when comparing current findings to previous studies, our results fall partially in line with research by [17], who found a significant association between fine motor skills (FMSs) and narrative abilities in preschool-aged children. Winter et al.’s hierarchical regression analyses revealed that FMSs accounted for 12% of unique variance in narrative comprehension when added to models that already included language skills. In comparing our findings to previous research, it is essential to note that our study utilized a comprehensive approach by examining both gross and fine motor skills within the context of narrative comprehension. This difference may lead to varying degrees of impact on narrative comprehension, as each type of motor skill engages different cognitive processes.

Additionally, by adopting a multicomponent model, we aim to illustrate the interplay among various cognitive skills, emphasizing that motor skills contribute not only to physical development but also to the cognitive processes underlying narrative comprehension. Therefore, although our results are consistent with the idea that motor skills are relevant to narrative comprehension, the unique characteristics of the tasks and motor skills in each study suggest that further research is needed to delineate these relationships more clearly. Addressing these differences will be critical in future investigations to enrich our understanding of how fine and gross motor skills interact in the context of cognitive and language development.

What we know from previous studies is that motor skills are relevant in the consolidation of cognitive skills during the early stages of development. Better motor skills are associated with improved cognitive control and allow for the development of cognitive flexibility, as well as a greater ability to remember and manipulate information present in the environment. Finally, movement, motor activities, and environment exploration lead to better understanding of others and, therefore, strengthen the ToM. At the same time, the engagement of motor skills not only facilitates the physical exploration of the narrative environment but also fosters imaginative processes, enabling children to mentally visualize scenes, characters, and actions described in the narrative. Exploring the body and physical environment through movement can stimulate imagination and encourage the formation of vivid mental images while producing or listening to a story [41]. This active involvement with the narrative content enhances comprehension by creating a richer and more immersive storytelling experience. These findings align with the principles of embodied cognition, which propose that cognitive processes are fundamentally rooted in bodily experiences and sensorimotor interactions. This perspective implies that cognitive and physical experiences are intrinsically linked, making it challenging to study cognitive abilities independently of motor abilities. Our results highlight that language acquisition, particularly narrative comprehension, involves a sensorimotor component [12,17]. Research has indicated that motor skills play a role in the development of narrative comprehension. One possible explanation may be that motor skills influence children’s interaction with the physical environment, enabling active exploration and experimentation with different actions. This interaction could foster higher-order skills [1], including narrative understanding, by promoting familiarity with spatial relationships, temporal sequences, and causal connections within a story. In addition, motor skills appear to support the ToM and executive functions such as WM and cognitive flexibility, which are key components for understanding complex narratives, supporting children’s holistic growth and providing clues in favor of embodied cognition. Movement-based exploration of one’s body and physical environment can also stimulate the imagination, enabling children to form vivid mental images as they listen to or produce stories [41], thus enriching narrative comprehension through visualization of scenes, characters, and actions. In summary, motor skills can provide a scaffolding function for the development of cognitive and linguistic skills critical to narrative comprehension. 

Overall, our results provide new, albeit preliminary evidence, that incorporating motor skills into multicomponent models of narrative comprehension provides a more comprehensive understanding of the cognitive processes involved in interpreting and internalizing stories. Given the wide developmental span from 3 to 6 years, we selected tasks that were appropriate across this age range and used standardized scores where available. Age was statistically controlled in all models, yet we approached the interpretation of the results with caution, acknowledging that the observed differences may reflect both individual variability and developmental shifts in cognitive and motor abilities. Future studies could benefit from narrower age bands to better capture age-specific trajectories and disentangle age-related versus individual effects.

There is another important point that deserves further discussion. Given the cross-sectional nature of this exploratory study, we did not test the mediation effect of cognitive skills in the relationship between motor skills and narrative comprehension, although we tested and found indirect pathways. These results offer initial insights that warrant further investigation. It would be important to investigate the mediation effect of cognitive skills in the relationship between motor skills and narrative comprehension, which may be examined through longitudinal studies. Future longitudinal studies will be crucial for better understanding the causal mechanisms at play and determining whether cognitive skills fully mediate the motor-to-narrative relationship. Continuing this line of research is essential for providing more conclusive evidence. Furthermore, our study serves as a valuable starting point, offering intriguing insights and providing a solid foundation for future research. As mentioned in the introduction, motor skills may have a different influence on different developmental stages, and this should be accounted for in further investigations.

A second limitation of this study is the use of a global motor skills score rather than separate fine- and gross motor skill measures. This choice was driven by the exploratory nature of our study and our interest in examining motor skills within the multicomponent model. However, future research should investigate the distinct contributions of fine and gross motor skills to narrative comprehension, as they may play different roles in this process.

Another important limitation concerns the model fit, particularly the RMSEA value, which is in the moderate-to-high range and at the threshold of acceptability. This suggests that while our model provides valuable insights, there is room for improvement in future studies. Given that this study represents an initial step in exploring the relationship between motor skills and narrative comprehension, future research should aim to refine these models by increasing sample sizes and using more sensitive measures. An additional avenue for future research involves examining whether motor skills exert an even stronger influence on narrative production compared to comprehension [17]. Given that narrative production requires not only understanding but also organizing and expressing complex information, it is plausible that motor abilities play a role in supporting these processes. Furthermore, future studies could integrate motor and narrative tasks within the same experimental paradigm. We are optimistic that a larger sample and longitudinal data will help resolve some of the uncertainties raised in this initial investigation, allowing for a more comprehensive understanding of the relationship between motor skills and narrative comprehension. This progression will also enable us to further explore the potential of embodied cognition in language development.

## Figures and Tables

**Table 1:** Descriptive statistics. As can be seen, children’s performance across all tasks covered a large range of scores, and none showed ceiling effects.

	1st Qu.	Mean	3rd Qu.	Std.Dev.	Median	Min	Max	Skew	Kurtosis
Narrative comprehension	6	7.03	9	2.19	7.50	0	10	−0.85	0.58
Motor skills	192.25	201.64	211	13.71	204.00	167	240	−0.14	0.15
CS ToM	3	5.93	8	3.10	5.00	1	12	0.33	1.07
CS WM	11	12.63	14	2.27	13	6	16	−0.87	0.34
CS Flexibility	6	13.97	18	6.04	18.00	5	24	−0.44	1.57
Language skills	5	6.97	8.75	2.31	7	1	12	−0.17	−0.53
Age	45.25	54.69	62.75	10.21	54.00	34	73	0.02	1.11

**Table 2:** The correlation matrix.

	Narrative Comprehension	Motor Skill	CS ToM	CS WM	CS Flexibility	Language Skill
Narrative comprehension	-					
Motor skills	0.12	-				
CS ToM	0.46 ***	0.26 *	-			
CS WM	0.35 **	0.11	0.45 ***	-		
CS Flexibility	0.54 ***	0.21	0.61 ***	0.36 **	-	
Language skills	0.54 ***	0.08	0.56 ***	0.45 ***	0.57 ***	-
Age	0.58 ***	−0.13	0.42 ***	0.34 **	0.55 ***	0.53 ***

* *p* < 0.05, ** *p* < 0.01, *** *p* < 0.001.

**Table 3:** Partial correlation matrix controlled by age.

	Narrative Comprehension	Motor Skill	CS ToM	CS WM	CS Flexibility	Language Skill
Narrative comprehension	-					
Motor skills	0.23	-				
CS ToM	0.29 *	0.32 **	-			
CS WM	0.20	0.16	0.35 **	-		
CS Flexibility	0.33 **	0.36 **	0.47 ***	0.22	-	
Language skills	0.35 **	0.15	0.41 ***	0.34 **	0.37 **	-

* *p* < 0.05, ** *p* < 0.01, *** *p* < 0.001.

**Table 4:** Bootstrap parameter estimates.

Bootstrap										
lhs	rhs	est	se	z	*p* Value	CI.Lower	CI.Upper	Std.Lv	Std.All	Std.Nox
Cognitive skills	Motor skills	0.006	0.002	3.062	0.002	0.002	0.01	0.006	0.448	0.012
Cognitive skills	Age in months	−0.001	0.007	−0.166	0.868	−0.018	0.012	−0.001	−0.026	−0.003
Cognitive skills	Language skills	0.195	0.042	4.636	0	0.107	0.274	0.195	0.409	0.415
Narrative skills	Cognitive skills	0.558	0.228	2.444	0.015	0.146	1.016	0.558	0.293	0.293
Narrative skills	Motor skills	0.01	0.003	3.071	0.002	0.004	0.017	0.01	0.415	0.011
Indirect effect through CSs		0.003	0.002	1.791	0.073	0.001	0.008	0.003	0.131	0.004

## Data Availability

The original contributions presented in the study are included in the article, further inquiries can be directed to the corresponding author, subject to privacy and data protection constraints.
